# Fluxer: a web application to compute, analyze and visualize genome-scale metabolic flux networks

**DOI:** 10.1093/nar/gkaa409

**Published:** 2020-05-22

**Authors:** Archana Hari, Daniel Lobo

**Affiliations:** Department of Biological Sciences, University of Maryland, Baltimore County, Baltimore, Maryland 21250, USA; Department of Biological Sciences, University of Maryland, Baltimore County, Baltimore, Maryland 21250, USA

## Abstract

Next-generation sequencing has paved the way for the reconstruction of genome-scale metabolic networks as a powerful tool for understanding metabolic circuits in any organism. However, the visualization and extraction of knowledge from these large networks comprising thousands of reactions and metabolites is a current challenge in need of user-friendly tools. Here we present *Fluxer* (https://fluxer.umbc.edu), a free and open-access novel web application for the computation and visualization of genome-scale metabolic flux networks. Any genome-scale model based on the Systems Biology Markup Language can be uploaded to the tool, which automatically performs Flux Balance Analysis and computes different flux graphs for visualization and analysis. The major metabolic pathways for biomass growth or for biosynthesis of any metabolite can be interactively knocked-out, analyzed and visualized as a spanning tree, dendrogram or complete graph using different layouts. In addition, *Fluxer* can compute and visualize the *k*-shortest metabolic paths between any two metabolites or reactions to identify the main metabolic routes between two compounds of interest. The web application includes >80 whole-genome metabolic reconstructions of diverse organisms from bacteria to human, readily available for exploration. *Fluxer* enables the efficient analysis and visualization of genome-scale metabolic models toward the discovery of key metabolic pathways.

## INTRODUCTION

Metabolic reconstructions from whole-genome sequencing and biochemical data aim to determine all metabolic processes occurring within a cell or whole organism, which can then be integrated into genome-scale metabolic models (GEMs) able to predict cellular phenotypes (1,2). Constraint-based analytical methods such as Flux Balance Analysis (FBA) can be applied to such models to predict the steady-state metabolic fluxes during cellular growth (3) and trace the metabolic flux from input nutrients to biomass growth and output metabolites (4). Several software tools have been developed for performing FBA (5,6). However, the analysis and visualization of the resultant mathematical solutions is a current challenge due to the large number of reactions that a typical GEM contains. This hinders our ability to identify the global metabolic flux through all reactions in the network and understand how the different metabolites participate in different growth phenotypes. The identification of key chemical routes between metabolites of interest, biomass growth and output bioproducts toward metabolic engineering applications requires novel user-friendly pathway analysis tools able to process and efficiently visualize genome-scale models with thousands of reactions and metabolites.

Several tools currently exist for the analysis and visualization of GEMs. The Systems Biology Markup Language (SBML) (7) is the standard format to specify and store GEMs. Cytoscape is a popular network visualization and analysis desktop tool (8,9), for which several plugins are available for processing SBML metabolic models (10,11). However, the resultant networks including all the reactions and metabolites in a GEM are typically a ‘hairball’ of reactions, making it difficult to visualize and understand individual pathways. The visualization tools ModelExplorer (12) and Grohar (13) for metabolic networks have a similar limitation when visualizing complete GEMs. Escher (14) is one of the most widely used tools for GEM interactive visualization, with an easily accessible web-based interface. However, Escher only displays a sub-set of reactions in the model, using predefined maps to position pre-selected metabolites and reactions. A current extension (15) is also able to perform FBA through the Escher web application, but is still limited to the subset of metabolites and reactions predefined in the layout map. Similarly, Pathview (16) and its web implementation (17) can visualize metabolic pathways for GEMs using predefined layout maps for a subset of reactions, which are obtained from KEGG (18). MetExplore (19) is a web application able to import and explore GEMs and visualize them with the MetExploreViz component (20). However, the application is optimized for visualizing small subsets of reactions and is not suitable for visualizing whole GEMs with an automated clean layout that is easy to explore. There is thus a need for user-friendly tools based on robust graph-theory methods (21–24) that can automatically visualize with efficient and clean layouts a complete GEM and its FBA solution fluxes directly from an SBML model.

Here we present Fluxer, a web-application for the automated computation of GEM graphs based on flux and their interactive visualization with a user-friendly interface. The application is able to take as input an arbitrary GEM specified in SBML format and automatically compute and efficiently visualize the complete network and their metabolic fluxes. The tool performs FBA of the model and includes several algorithms to compute optimized visualizations of complete GEMs, including spanning trees, dendrograms and physics-based force layouts. The application can also compute and display the *k*-shortest metabolic paths between two reactions or metabolites, using a user-customizable metric for the link weights based on the computed metabolic fluxes. The graph visualizations are interactive, and the user can select any metabolite or reaction as the root for the flux tree, obtain information about the reactions and metabolites such as their chemical structures, and easily adjust the node labels, weight calculations, and inclusion or not of cofactors, zero-flux reactions or cellular localizations in the visualized graph. Fluxer allows any user with no programming experience to exploit the potential of genome-scale metabolic models in their ability to assist in the understanding of the whole metabolic network of an organism and predict specific phenotypes. Fluxer’s combined use of FBA and graph theory for metabolic network visualization and analysis in a user-friendly web-application can pave the way for advancements in applications for metabolic engineering.

## FEATURES

Fluxer computes genome-scale metabolic flux networks, analyze and visualize them in the form of spanning trees, *k*-shortest paths and complete graphs with an interactive and user-friendly web interface. Any GEMs in SBML format can be uploaded to the tool, which then performs FBA optimization and calculates and renders the complete model with different interactive graph visualizations. The metabolic networks are encoded as bipartite graphs, where nodes represent metabolites or reactions and edges the involvement of a metabolite in a reaction as a product or a reactant. Nodes can be selected to display detailed information including metabolite structures and reaction fluxes. Furthermore, any number of reactions can be knocked-out through the interface to simulate enzyme gene deletions and their phenotypic effects in terms of growth rates and fluxes. The different available graph representations make use of the computed steady-state reaction fluxes, which together with the stoichiometric coefficients and molecular weights constitute the weight of each edge. The spanning tree graph calculates the input flux pathways toward the root node—the biomass growth or any other reaction or metabolite of interest—visualizing the most important pathways contributing to the root node. The *k*-shortest paths graph calculates the shortest pathways with highest fluxes between two metabolites or reactions of interest, easily visualizing each separate path in an interactive graph. Finally, the complete graph includes all the reactions and metabolites of the model, which can be visualized as a reaction network or as a tree rooted with a particular metabolite or reaction of interest. The models uploaded to the application can be privately shared with an automatically generated unique web link, and >80 genome-scale metabolic reconstructions from the BiGG Models knowledge base (25,26) are readily available in the web server for their analysis and visualization. The web application includes a tutorial explaining the main analysis options and features of the tool.

### Spanning tree

A GEM can contain thousands of metabolites participating in thousands of reactions, which represents a challenge for their complete visualization and understanding. The most important pathways leading to a given metabolite or reaction can be buried in the complexity of a whole-genome metabolic network. Most current network visualization tools overcome this topological problem by dividing the network into different submodules and either requiring the users to manually place the nodes or have pre-defined maps of the most popular pathways. However, manually placing nodes in a neat layout is a time-consuming task and when dividing the whole metabolic network into submodules, it is difficult to have a complete and global understanding of the main pathways leading to a given metabolite.

Fluxer is able to automatically and efficiently visualize any GEM as a spanning tree rooted with the objective function reaction defined in the model (usually the biomass growth reaction) or any other reaction or metabolite of interest. The spanning tree is recursively connected with the upstream most important reactions and metabolites leading to the root to neatly visualize the whole network. Each metabolic input and output for each reaction (an edge in the graph) is assigned a weight, which is the product of a user-selectable combination of the reaction metabolic flux (calculated from the FBA analysis), stoichiometry coefficient and metabolite molecular weight. The tree includes every node (metabolite or reaction) in the model once, without duplication. Starting from the root node, the algorithm builds the tree by iteratively adding the edge with the highest weight that connects a current node in the tree with a node not yet present in the tree; in case of more than one candidate edge having the same weight, the edge with the shortest path to the root node is selected; and in case of similar depth, the edges are selected according to the alphabetical order of their labels. In addition, precedence is always given to the edges with fluxes toward the root node, so the elements participating in the production of the metabolite or reaction of interest are always added first to the tree. In this way, the selected edge weights determine the layout of the tree. By default, the weights are calculated as the product of the reaction metabolic flux and metabolite stoichiometry coefficient, so the metabolites selected first are those that contribute the most amount (or mass if the molecular weight is also selected) to the root metabolite or reaction. If none of the weight options are selected, all edges would have a default weight of 1 unit, and the spanning tree algorithm is equivalent to an alphabetically sorted breadth-first search, showing the shortest metabolic paths to the root node from every metabolite and reaction in the model.

Five different layouts can be used to visualize the resulting spanning tree. A tree layout based on Reingold and Tilford’s tidy drawing algorithm (27) shows the nodes in hierarchical order, where the leaf nodes of the tree are placed on the left of the graph and the root node is placed on the right. In this way, the graph shows the metabolic flux toward the root node from left to right. A dendrogram layout can be selected to show all the leaf nodes at the same level in the left side of the graph, so they are easily identified, whereas the root node is placed in the right location of the graph. In addition, the tree and dendrogram layouts can be visualized with a radial configuration, useful for a more compact representation. Finally, a physics-based force layout simulation based on a velocity Verlet numerical integrator for simulating particles motion dynamics (28) can be selected to display the spanning tree. In this force layout, nodes dynamically repeal each other with repulsive forces, whereas edges keep them connected. This results in a dynamic arrangement of the position of the nodes, which tends to converge to organic configurations where related metabolites and reactions are clustered together.

**Figure 1.**
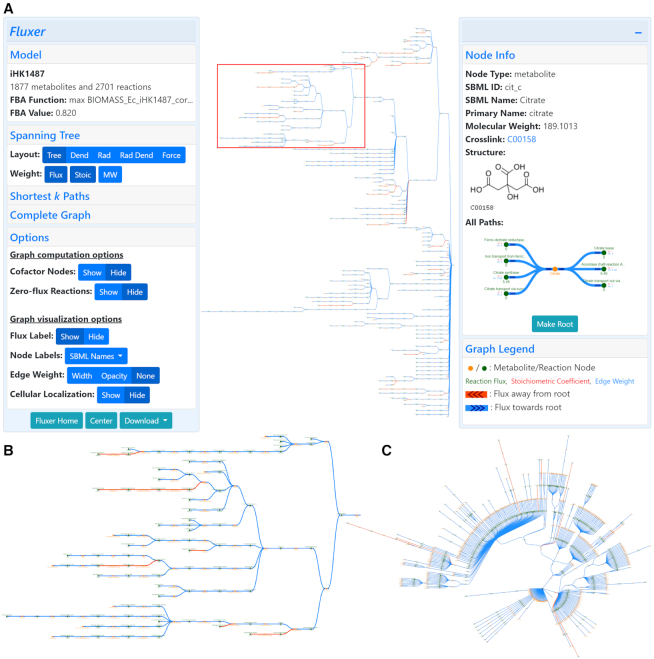
Fluxer interface showing a spanning tree visualization of a genome-scale metabolic model of *Escherichia coli* BL21 (DE3) rooted in the cell biomass reaction. (**A**) The interactive web application can display the complete metabolic network with clear layouts computed in base of the optimized metabolic fluxes. The left card shows general information about the model and gives access to different visualization options. The right card shows details about the selected metabolite or reaction. (**B**) Zoomed view of one of the branches in the metabolic flux tree (red box). (**C**) Radial visualization of the flux tree including cofactor metabolites. Green nodes represent reactions and yellow nodes represent metabolites. The reaction flux is displayed below each reaction node. Arrow heads indicate direction of fluxes going toward (blue edges) or coming from (red edges) the reaction or metabolite selected as the root of the spanning tree.

The spanning tree with the different layouts can be visualized in Fluxer with an interactive user-friendly interface, which allows the exploration and customization of the different options for the graph and layout computations. Figure 1A shows the web application interface displaying the spanning tree for a GEM of *Escherichia coli* BL21 (29), excluding reactions with zero flux and cofactor metabolites. The tree is rooted with the cell biomass reaction, which represents the objective function in the FBA. The FBA optimization resulted in a growth rate of 0.820 h^−1^, as shown in the interface—a value identical to the original report (29). The left card also shows various graph options including layouts, weight metrics and other general customizations. The right card displays the chemical structure, names, id, crosslink and reactions and metabolites of the selected node. Figure 1B shows the zoomed region in the graph top area marked with the red box in Figure 1A, which includes reactions leading to the biosynthesis of citrate for energy production during glycolysis and the citric acid cycle. These pathways were reported to carry the highest flux in the organism (29), and Fluxer placed them at the top of the graph as the most important pathways for the selected tree root—the cell biomass reaction for this model. Figure 1C shows the spanning tree with a radial layout when including the cofactor metabolites, which results in a more compact visualization useful for large graphs. These results show how Fluxer can categorize and recognize the most important pathways in a GEM by combining the computed fluxes with the spanning tree algorithm to display on the top area of the visualization the reactions and metabolites highly contributing to the root node selected for the tree.

### *K*-shortest paths

Finding the most important metabolic pathways between two metabolites of interest can provide insights into the metabolism of the cell and aid in their optimization for metabolic engineering applications, as well as for the formulation of therapeutic strategies in metabolic-based disorders (23,24,30). A number of computational approaches have been proposed for metabolic pathfinding (31). Among them, the *k*-shortest path method is a popular approach to determine the most important and biologically meaningful metabolic pathways by finding the shortest *k* metabolic paths between two metabolites given a particular metric (32). Several tools are available for metabolic pathfinding, but they are generally restricted to pre-processed GEMs, not freely-available, or lack an intuitive user-friendly graphical interface (33,34).

Fluxer can compute and visualize the *k*-shortest paths between two metabolites or reactions for any GEM. The method is based on Yen’s algorithm to compute the *k*-shortest loop-less paths in a network (35). The algorithm first finds the shortest path between the two given nodes of interest. For this, the application uses the same method that for the spanning tree algorithm, where the edge weights can be selected to be a combination of the reaction metabolic flux, stoichiometry coefficient and molecular weight. Next, the algorithm iteratively removes every edge in the shortest paths found so far, one at a time, after which the same shortest path algorithm is applied. The computation stops when the first *k* shortest paths have been found or all edge removals have been exhausted. Alternatively, the algorithm can find the best *k*-shortest paths by exhausting all edge removals and then returning the *k* best paths among all found, which are guaranteed to be optimal at the expense of a longer computational time. Similar to (30), cofactor nodes—including currency metabolites and small molecules like water and metabolic factors like ATP—can be excluded from the computation of the shortest paths, since they are involved in a large number of reactions that are not connected by any other metabolite. In this way, the algorithm finds the most important pathways in terms of number of reactions and flux between two metabolites or reactions of interest.

The resulting *k*-shortest paths can be visualized together as an acyclic graph with a dagre or a force layout. The dagre layout (Chris Pettitt) positions the nodes of the acyclic graph using layered techniques and cross-minimization algorithms (36–39), neatly separating the different paths found by the algorithm. In addition, the *k*-shortest paths can be visualized with the simulation-based force layout, similar to the method in the spanning tree visualization. This layout is useful for large *k*-shortest path graph, which highlights clustering in the graph.

**Figure 2.**
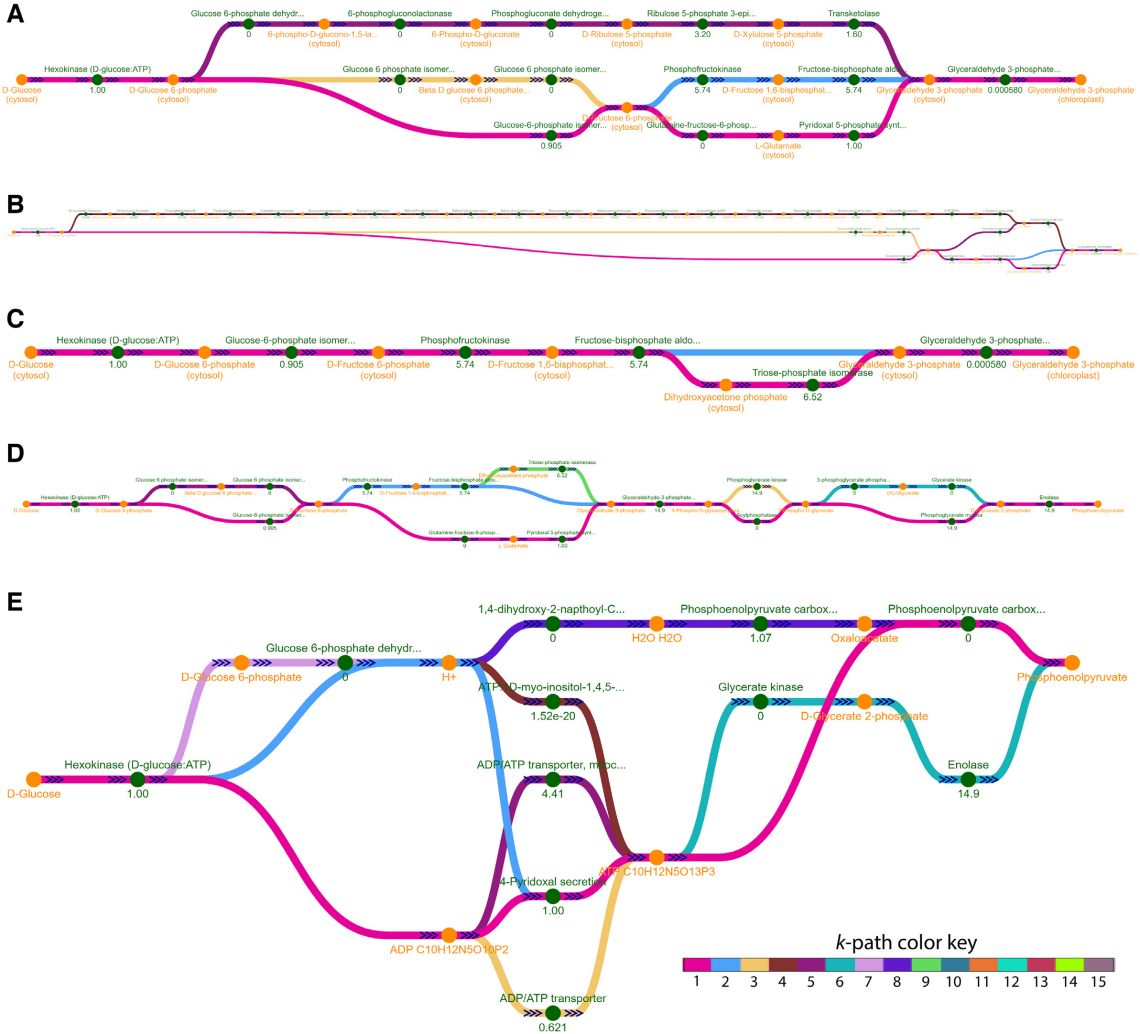
Computation and visualization by Fluxer of different best *k*-shortest paths in a metabolic model of *Plasmodium falciparum* 3D7. Each path is displayed with a different color; when overlapping, the shorter path color is shown. (**A**) The computed five shortest paths between d-glucose and glyceraldehyde 3-phosphate using the number of reactions as a metric. (**B**) Changing the metric to be the product between the reaction flux and the metabolite stoichiometric coefficients results in longer paths but with higher fluxes. (**C**) Excluding the zero flux reactions results in only two paths carrying flux between the source and target metabolites. (**D**) The ten shortest paths between d-glucose and phosphoenolpyruvate using the number of reactions as a metric. (**E**) When including the cofactor metabolites in the graph computation, the ten shortest paths using the number of reactions as a metric contain fewer reactions.

A set of *k*-shortest path graphs computed and visualized with Fluxer for a GEM of *Plasmodium falciparum* 3D7 (40) from the BiGG Models knowledge base (26) is presented in Figure 2. All the graphs shown are visualized with the dagre layout. Figure 2A shows the five shortest paths between d-glucose and glyceraldehyde 3-phosphate using just the number of reactions as a metric. The figure illustrates how each path is shown with a different color (when overlapping, the color of the shorter path is shown). Reactions from both the glycolysis and the pentose phosphate pathways are involved in the shortest paths between the two metabolites selected, as it was predicted previously (41). In addition, Fluxer can clearly display fluxes and reactions between compartments in the model, as it is shown in the transport reaction of glyceraldehyde 3-phosphate from the cytosol to the chloroplast (last reaction). Figure 2B shows the shortest paths obtained when the edges are weighted with the product between the reaction flux and the metabolite stoichiometric coefficients. In this case, the resulting graph shows at the top the *myo*-inositol biosynthesis pathway, as it is indeed an alternate route when glucose cannot be converted to fructose 6-phosphate, as previously found in *P. falciparum* (42). The reactions that do not carry flux can be excluded from the shortest path calculation, as shown in Figure 2C. Finally, Figure 2D shows the best 10 shortest paths computed between d-glucose and phosphoenolpyruvate with the number of reactions as a metric. When the cofactor metabolites are included in the computation of the 10 shortest paths, the computed pathways are shortened considerably, as shown in Figure 2E, due to the reactions being connected by small molecules such as hydrogen ions and water, or cofactors such as ATP. Thus, Fluxer shortest path graph can be used to discover alternative pathways between metabolites in a GEM through the use of flux values and stoichiometric coefficients in the metric, representing an essential tool for metabolic engineering.

### Complete graph

The spanning tree and *k*-shortest path graphs can be used to analyze a GEM and discover the most important reactions and metabolites for different applications. However, it is also useful to display the complete GEM including all the reactions and metabolite connections, which can highlight global reaction and metabolite clusters in the model. For this, Fluxer can visualize the complete graph with all the reactions and metabolites in the GEM using the same tree and physics-based force graph layouts as for the spanning tree graph. The complete graph can be visualized with tree layouts rooted in any metabolite or reaction in the model, which includes all the reaction-metabolite edges. The tree layouts of the complete graph are computed with a breadth-first search, and in contrast to the spanning tree, it includes duplications of metabolite and reaction nodes so all edges are displayed in the graph. In addition, Fluxer can visualize the complete graph with the layouts information directly included in GEMs containing the SBML Layout Extension (43).

**Figure 3.**
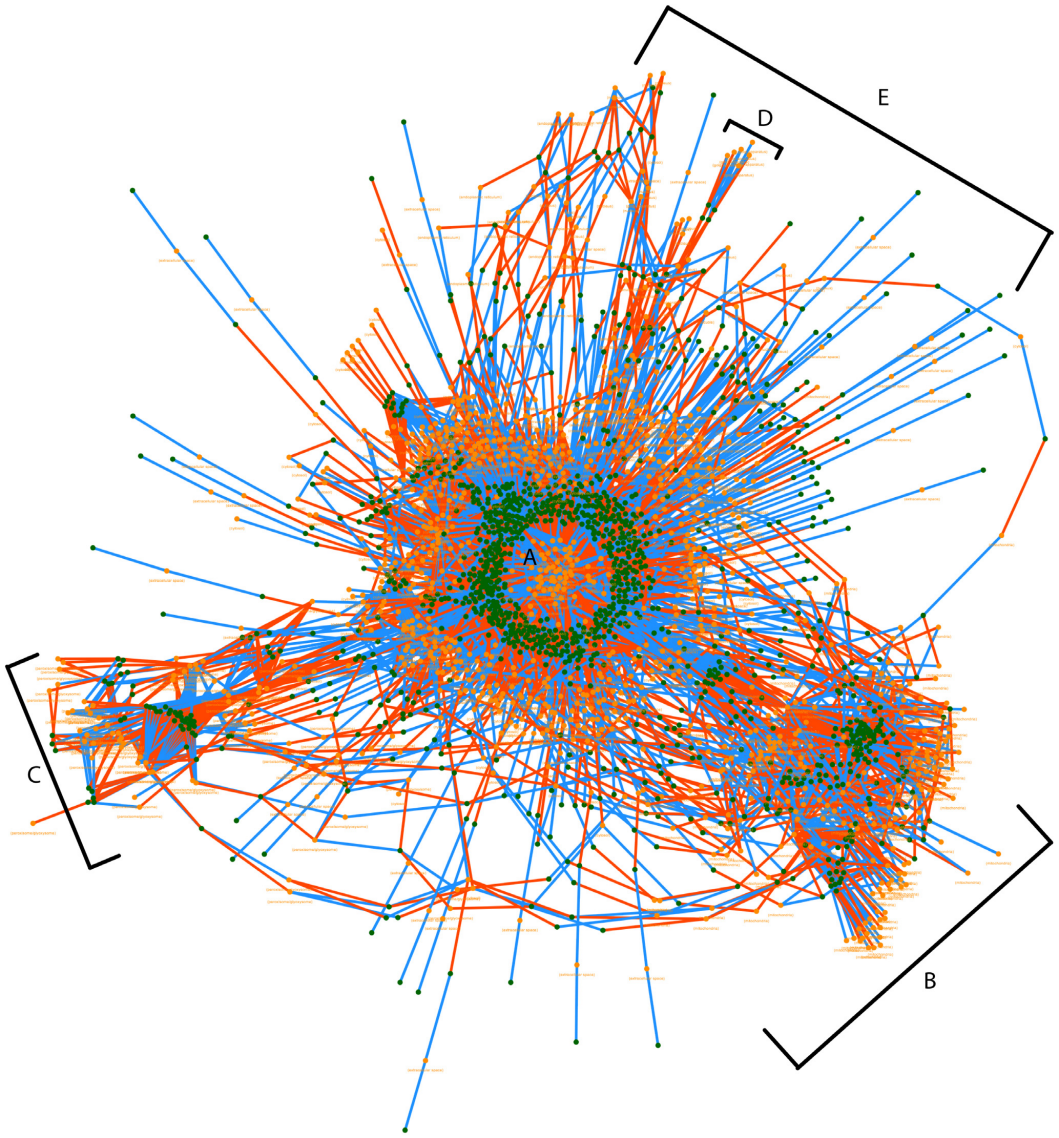
Final configuration of a physics-based force layout rendered by Fluxer for the compete graph of a metabolic model of *Saccharomyces cerevisiae* S288C. The metabolites and reactions of the whole-genome network are automatically clustered according to their cellular localization. The labels indicate the main resulting cluster, each corresponding to a particular cellular localization. (**A**) cytosol, (**B**) mitochondria, (**C**) peroxisome, (**D**) cell nucleus and (**E**) endoplasmic reticulum. Red edges indicate product-reaction node connections, while blue edges indicate reaction-reactant node connections. The cellular localization of the metabolites and reactions are displayed below each node.

The complexity of GEMs is evident when viewing the complete model in a force-based layout. Figure 3 shows the complete graph of a fully compartmentalized GEM for *Saccharomyces cerevisiae* S288C (44) using the force layout in Fluxer. Interestingly, the layout results in the metabolite and reactions in the model forming clusters according to their location in the cell. The largest cluster is centered in the graph (A) and contains mostly cytosol associated metabolites and reactions. These nodes are the closest to the cell biomass reaction. In addition, four other clusters are formed with metabolites and reactions associated with different cellular localizations, as they are visible in the resulting layout. These clusters include metabolites and reactions taking place in the mitochondria (B), peroxisome (C), cell nucleus (D) and endoplasmic reticulum (E). Although the complexity of the complete graph with the force layout prevents a tidy representation of a complete metabolic network, it is useful for finding global clusters and trends, as shown in this example.

### User interface

The user-friendly and interactive web interface of Fluxer allows the upload of SBML models, optimization and knockout of its reactions, selection of the different graph algorithms and layouts to visualize, their customization, navigation through the graphs, and the display of detailed information of the model metabolites and reactions (Figure 1). A collapsible card gives access to the different options of the algorithms and displays information about the model, such as name, objective function and flux optimized value. Another collapsible card displays information about the metabolites and reactions, such as names and IDs, molecular weight and flux, metabolite structures and includes external links to KEGG (18) or MetaNetX (45). The graph itself can contain labels to dynamically display different information about the nodes, such as metabolite and reaction names, fluxes and localizations. In addition, the opacity or width of the edges can be selected to represent their weights, resulting in a global clear visualization of metabolic flux. Metabolite and reaction nodes can be clicked to show their detailed information, as well as be selected as root for the different tree layouts or be knocked-out in the flux optimizations. The information card also contains a mini graph showing all the metabolites participating in the selected reaction or all the reactions that the selected metabolite is involved in. The interface includes options to exclude from the computed graphs the reactions with zero flux as well as the cofactor metabolites. Fluxer is compatible with all major browsers in desktop and mobile devices and offers an exporting function to download high-quality raster and vector images of all the graphs generated.

## IMPLEMENTATION

Fluxer is implemented in Python using Flask (Pallets) for the backend, HTML5 and JavaScript for the frontend, and SQLite for the database. FBA optimization is performed with COBRApy (46) and the graph layouts use the D3.js library (47). Reaction and metabolite naming conventions for GEMs is a current challenge in the field (48), and Fluxer pools information from ModelSEED, BiGG and MetaNetX (25,26,45,49) to identify molecular weight, crosslinks, chemical formula, and other metabolite and reaction information for the GEMs uploaded through the interface. The list of metabolites considered cofactors was based on (30). Any SBML file compatible with COBRApy can be uploaded, optimized and visualized with the web application. Fluxer is able to analyze and display graphs for GEMs even as large as the human metabolic model RECON3D (50), containing over 5000 metabolites and 10 600 reactions.

## DISCUSSION

We presented here Fluxer, a user-friendly web application for the computation, analysis and visualization of flux graphs for GEMs. The tool can perform FBA including specific reaction knockouts and compute metabolic network representations based on spanning trees, *k*-shortest paths and complete graphs. The different networks can be visualized with tree, dendrogram, radial, dagre and force-based layouts. The web application allows any user to load models in SBML format and interact and customize the different metabolic networks generated. Users require no special training or software installations to access and use the web application. The ability of Fluxer to perform visual comparisons of reaction fluxes makes it a powerful tool for understanding metabolic phenotypes and discovering pathways for metabolic engineering applications.

While Fluxer computes graph visualizations centered on a particular root metabolite or reaction of interest, other approaches have been proposed for the global analysis of GEMs. Extreme pathways (51) represent the set of steady state fluxes that lay at the border of the solution space given by the model constraints. As such, they are useful to analyze the full capability of a metabolic model. Elementary flux modes (52) are the smallest subset of reactions that allow the system to operate in steady state, giving important insights into the most essential pathways within a metabolic model. The MinSpan algorithm (53) can decompose complete genome-scale models into their most independent pathways. In this way, it serves as an efficient method to understand the pathway structures that a whole model contains. In contrast, the spanning tree computed in Fluxer is focused on finding the most important pathways that contribute to a single root metabolite or reaction by considering the fluxes obtained from a particular FBA solution. Indeed, the spanning tree computed by Fluxer could be combined in future work with these global model analyses to efficiently visualize their resultant pathway structures.

Further future work will extend the current functionality of Fluxer. The capabilities to perform FBA customizations beyond the knockout of reactions will be improved to include different optimization objectives and the ability to change the flux boundaries of any reaction—particularly to specify different uptake rates for different growth media. New graph layouts will be implemented, such as maps to highlight the metabolic composition of a particular phenotype state. Finally, the web server will include the possibility for the user to make public any model uploaded to the application, and then list them in a dynamically updated repository of genome-wide metabolic models.

Fluxer provides not only a topological solution for visualizing genome-scale metabolic models, but also specific computational methods to analyze reaction networks and fluxes. The comparison of metabolic network connectivity and motifs across organisms can provide evolutionary insights (54), an approach that could be enhanced by examining metabolic flux networks such as those computed by the presented application. User-friendly tools, such as Fluxer, are able to analyze complex datasets, networks and mechanistic models (55,56), together with novel visualization and encoding software applications (57–59), and will be essential for the understanding of complex biological mechanisms (60–62) and the discovery of novel phenotypes (63,64). In conclusion, Fluxer represents a user-friendly resource to visualize and analyze genome-scale flux networks toward the global understanding of whole-organism metabolism and the advancement of the much sought-after applications in metabolic engineering.
